# Viviparity and habitat restrictions may influence the evolution of male reproductive genes in tsetse fly (*Glossina*) species

**DOI:** 10.1186/s12915-021-01148-4

**Published:** 2021-09-23

**Authors:** Grazia Savini, Francesca Scolari, Lino Ometto, Omar Rota-Stabelli, Davide Carraretto, Ludvik M. Gomulski, Giuliano Gasperi, Adly M. M. Abd-Alla, Serap Aksoy, Geoffrey M. Attardo, Anna R. Malacrida

**Affiliations:** 1 Department of Biology and Biotechnology, University of Pavia, Pavia, Italy; 2 Institute of Molecular Genetics IGM-CNR “Luigi Luca Cavalli-Sforza”, Pavia, Italy; 3 Research and Innovation Centre, Fondazione Edmund Mach (FEM), San Michele all’Adige, Italy; 4 Center Agriculture Food Environment (C3A), University of Trento, Trento, Italy; 5 Insect Pest Control Laboratory, Joint FAO/IAEA Programme of Nuclear Techniques in Food & Agriculture, Vienna, Vienna, Austria; 6 Department of Epidemiology of Microbial Diseases, Yale School of Public Health, New Haven, CT USA; 7 Department of Entomology and Nematology, University of California, Davis, Davis, CA USA

**Keywords:** *Glossina*, Male accessory gland genes, Testis genes, Habitat, Viviparity, Selective pressure

## Abstract

**Background:**

*Glossina* species (tsetse flies), the sole vectors of African trypanosomes, maintained along their long evolutionary history a unique reproductive strategy, adenotrophic viviparity. Viviparity reduces their reproductive rate and, as such, imposes strong selective pressures on males for reproductive success. These species live in sub-Saharan Africa, where the distributions of the main sub-genera *Fusca*, *Morsitans*, and *Palpalis* are restricted to forest, savannah, and riverine habitats, respectively. Here we aim at identifying the evolutionary patterns of the male reproductive genes of six species belonging to these three main sub-genera. We then interpreted the different patterns we found across the species in the light of viviparity and the specific habitat restrictions, which are known to shape reproductive behavior.

**Results:**

We used a comparative genomic approach to build consensus evolutionary trees that portray the selective pressure acting on the male reproductive genes in these lineages. Such trees reflect the long and divergent demographic history that led to an allopatric distribution of the *Fusca*, *Morsitans*, and *Palpalis* species groups. A dataset of over 1700 male reproductive genes remained conserved over the long evolutionary time scale (estimated at 26.7 million years) across the genomes of the six species. We suggest that this conservation may result from strong functional selective pressure on the male imposed by viviparity. It is noteworthy that more than half of these conserved genes are novel sequences that are unique to the *Glossina* genus and are candidates for selection in the different lineages.

**Conclusions:**

Tsetse flies represent a model to interpret the evolution and differentiation of male reproductive biology under different, but complementary, perspectives. In the light of viviparity, we must take into account that these genes are constrained by a post-fertilization arena for genomic conflicts created by viviparity and absent in ovipositing species. This constraint implies a continuous antagonistic co-evolution between the parental genomes, thus accelerating inter-population post-zygotic isolation and, ultimately, favoring speciation. Ecological restrictions that affect reproductive behavior may further shape such antagonistic co-evolution.

**Supplementary Information:**

The online version contains supplementary material available at 10.1186/s12915-021-01148-4.

## Background

The ability to secure mates and achieve fertilization is a fundamental measure of male reproductive success [1], and at the core of this is the ejaculate, which is responsible for inducing important post-mating responses in females [2, 3]. Traits and male reproductive genes involved in these post-copulatory interactions have been suggested to evolve rapidly due to sexual selection and sexual conflict [4–19]. This rapid evolution potentially leads to reproductive incompatibilities among lineages due to disruptions in male reproductive hybrid fitness [20–23]. Accelerated evolution in male reproductive genes is expected in viviparous insects, which invest considerable energy to produce a limited number of high-quality progeny. Indeed, viviparous reproduction reduces the reproductive capacity/rate, leading to increased inter- and intra-sexual conflict. This is the case of *Glossina* flies, sole members of the Glossinidae family (Schizophora, Calyptratae) and typical examples of K-strategists [24]. These flies reproduce through adenotrophic viviparity, which is defined as having intrauterine larval development and provision of all larval nourishment by the milk glands for the duration of its development [24–26]. This mode of reproduction has been associated with extreme morphological and functional adaptations [24, 27]. In these flies, a very important adaptive trait for fertilization is that the male ejaculate is encapsulated into a spermatophore, which is assembled within the female uterus from the secretions of the male accessory glands (MAGs) and testes during the later stages of copulation [28, 29]. The spermatophore functions as a protective container for the ejaculate, ensuring semen/sperm delivery directly to the female spermathecal ducts and inhibition of insemination by competing males [27, 29].

From an ecological point of view, tsetse flies are restricted to and distributed throughout sub-Saharan Africa, where they are the vectors of African trypanosomiasis in humans and animals [30–32]. Based on a combination of geographical distribution, behavioral, molecular, and morphological features, *Glossina* can be generally divided into three sub-genus groups of species: (i) *Morsitans*, largely savannah and woodland flies; (ii) *Palpalis*, riverine and lacustrine inhabitants, and (iii) *Fusca*, which are forest flies of West Africa [33]. The habitat restriction of each species group is an important and immediate determinant of their behavior [34]. It is conceivable that such habitat/ecoclimate restrictions could affect, at the population level, the number of interactions of males and females before, during and post-mating, thus impacting the intensity of intra- and intersexual conflict [35]. Indeed, different remating rates have been observed among members of the *Morsitans* and *Palpalis* groups [36–38].

It is still unknown whether the different levels of intra- and intersexual selective pressure potentially associated with habitat restrictions are reflected in *Glossina* lineage-specific patterns of evolution and positive selection of male reproductive genes. Here, we aim at exploring whether species with different habitat restrictions display different patterns of male reproductive gene evolution. To do so, we made use of the genome sequences of six species belonging to the *Fusca* (i.e., *Glossina brevipalpis*), *Morsitans* (i.e., *G. m. morsitans*, *G. pallidipes*, *G. austeni*), and *Palpalis* (i.e., *G. fuscipes*, *G. palpalis*) groups [30, 39], and the functional data obtained from the spermatophore proteome and the transcriptomes derived from the two male reproductive compartments, testes and MAGs, from *G. m. morsitans* [29].

We first derived a time-calibrated phylogeny of the six *Glossina* species to reconstruct their long evolutionary history. Next, we tested for evidence of positive selection in the male reproductive genes from the testes and MAGs. The data indicate that the overall evolutionary patterns of reproductive genes are consistent with the evolutionary history and the biology of the different *Glossina* lineages. Moreover, the evolutionary rate of genes from MAGs is faster than that of testes and is heterogeneous among and within the species groups.

## Results

### Time-calibrated phylogeny of *Glossina* species from the *Morsitans*, *Palpalis*, and *Fusca* groups

**Fig. 1:**
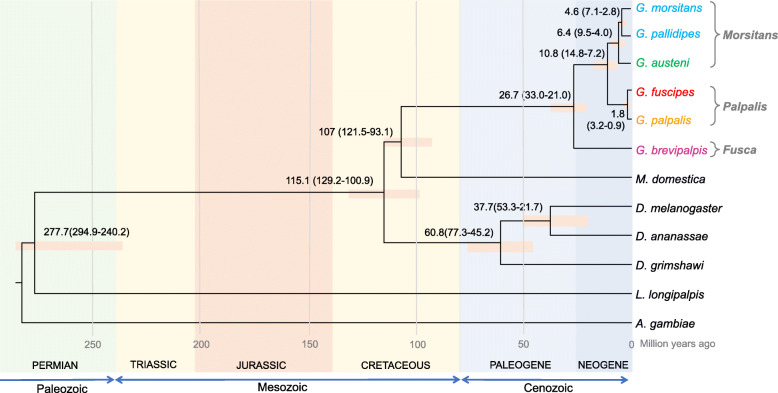
Genome-scaled phylogeny and divergence estimates. Bayesian consensus tree inferred on a dataset of 478,000 nucleotides by PhyloBayes using relaxed clocks and node/fossil constraints. Numbers at nodes are main divergence estimates and 95% High Posterior Densities, HPD (in parentheses) expressed in millions of years. Bars are the 95% HPD. All nodes received full support (100 Bootstrap support and 1.00 posterior probabilities, PP) in a Maximum Likelihood (RAxML) and Bayesian (PhyloBayes) analysis, except for the split of *M. domestica* from *Glossina* which had a PP of 0.96

As a premise to the analysis of the evolution of male reproductive genes in *Glossina*, a time-calibrated phylogeny of species within the *Morsitans*, *Palpalis*, and *Fusca* groups was obtained using *Musca domestica* (Calyptratae), three *Drosophila* species (Acalyptratae), *Lutzomyia longipalpis*, and *Anopheles gambiae* as outgroups (Fig. 1). This time-calibrated phylogeny, based on the previously developed genome-scaled data [30], suggests a mid-Cretaceous origin of the lineage leading to the *Glossina* genus (stem-group *Glossina*, mean of 107 million years ago (mya), posterior densities in Fig. 1) with an Oligocene radiation of extant *Glossina* species. *Glossina brevipalpis* diverged from the forest *Fusca* group at a mean of 26.7 mya. The divergence of the savannah *Morsitans* from the riverine *Palpalis* groups dated toward the middle Neogene (mean 10.8 mya). Within the *Morsitans* group, *G. m. morsitans*, *G. pallidipes*, and *G. austeni* diverged toward the end of the Neogene (mean 4.6 mya), while *G. austeni* split from the *Morsitans* group at 6.4 mya. The split of *G. fuscipes* from *G. palpalis* within the *Palpalis* group was the most recent with a mean of 1.8 mya. Based on these time-calibrated relationships, phylogeny-based tests of positive selection on male reproductive genes from testes and MAG body compartments were performed.

### Across the *Glossina* lineages the male reproductive genes from MAGs and testes are under different selective pressure

**Fig. 2:**
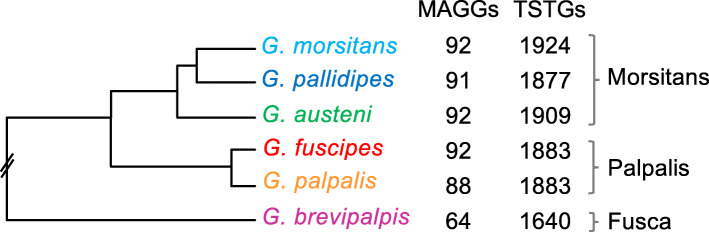
Evolution of TSTGs and MAGGs orthologs on the *Glossina* phylogeny. Number of genes orthologous to *G. m. morsitans* TSTGs and MAGGs for all considered *Glossina* species

A total of 5513 orthologous sequences (out of the 8088 considered) were detected across the six *Glossina* genomes (Table S1, S2). These orthologs were overlapped with a *G. m. morsitans* dataset of 2563 genes, of which 2436 display enriched transcription (≥ 5-fold) in the testes (TSTGs) and 127 in the MAGs (MAGGs) [29]. The overlapping orthologs consisted of 1924 TSTGs and 92 MAGGs and their species distribution is reported in Fig. 2. Due to its basal phylogenetic position, *G. brevipalpis* from the *Fusca* group shares the fewest orthologs (85% for TSTGs, 69% for MAGGs), probably due to high sequence divergence hindering a proper orthology assignation. It cannot be excluded, however, that this difference in the number of orthologs is also the result of a gain of TSTGs and MAGGs in the clade containing the other five species.

The nonsynonymous (*d*_N_) and synonymous (*d*_S_) substitution estimates on the MAGG and TSTG orthologs (92 and 1924, respectively), as well as for the all 5513 orthologs, indicate that the average level of selective pressure, measured by *d*_N_/*d*_S_, was significantly higher in MAGGs (0.183 ± 0.178 SD, var = 0.032) than in TSTGs (0.085 ± 0.068 SD, var = 0.005) and “all genes” combined (0.088 ± 0.071 SD, var = 0.005), which are used as a proxy of the genome-wide level (Wilcoxon test *P* < 10^−5^, for both comparisons). Moreover, there is a high heterogeneity in the selective pressure across MAGGs when compared to either TSTGs and “all genes” (Bartlett test of homogeneity of variances, K-squared = 196.97, df = 1, *P* < 10^−15^; and *K*-squared = 187.44, df = 1, *P* < 10^−15^, respectively), which is expected in presence of contrasting selective regimes (i.e., positive, purifying, and relaxed). In contrast, TSTGs were under a rate of molecular evolution similar, if not even more constrained, to the trend detected across the genome (Wilcoxon test *P* = 0.215; and Bartlett test of homogeneity of variances, *K*-squared = 6.721, df = 1, *P* = 0.010; Table S2).

**Fig. 3:**
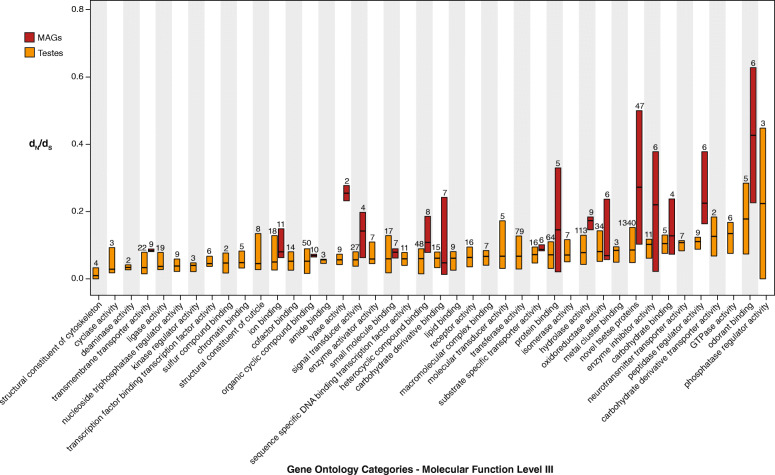
Mean *d*_*N*_*/d*_*S*_ ratio for tsetse genes grouped according to GO functional categories. GO functional categories (Molecular Function Level III) are reported for MAGGs and TSTGs (red and orange bars, respectively). Notched box plots show medians and extend to the first and third quartiles. Only categories with at least two members are shown. Numbers of genes assigned to each class is reported above the box plots

The heterogeneity in the selective pressure on MAGGs with respect to TSTGs is also reflected at the level of gene functional categories. Indeed, the 17 Gene Ontology (GO) categories of MAGGs display a *d*_N_/*d*_S_ average estimate of 0.2, ranging from a maximum estimate of 0.42 for “odorant binding” to a minimum value of 0.03 for “carbohydrate derivative binding” (Fig. 3; Table S1, S3). Heterogeneity for *d*_N_/*d*_S_ is also present within classes: odorant-binding proteins (OBPs) include six genes with *d*_N_/*d*_S_ from 0.12 (GMOY005875) to 0.73 (GMOY007314), and the novel tsetse protein (NTP) gene category [29], with estimates from < 0.1 (GMOY002769) to 1.22 (GMOY007759). The NTP category comprises genes that share no similarity to annotated sequences deposited in the GenBank database, as well as no recognizable domains based on BLAST analyses and structural homology searches (e.g., [40–43]. Conversely, the 42 GO functional categories of TSTGs displayed low heterogeneity and very low values in the *d*_N_/*d*_S_ average estimates (mean *d*_N_/*d*_S_ = 0.1), indicative of strong purifying selection. The most heterogeneous classes are OBPs and phosphatase regulators.

### The MAG and testes genes evolve differentially in the *Morsitans*, *Palpalis*, and *Fusca* lineages

**Fig. 4:**
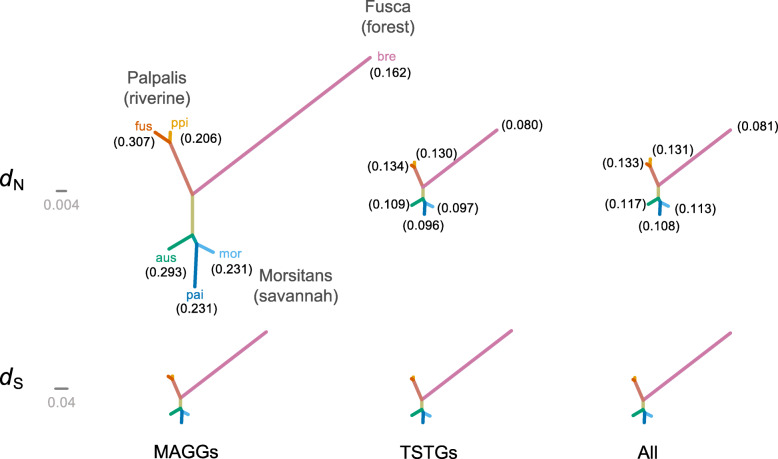
Consensus evolutionary analysis of orthologous genes in the six *Glossina* species. Upper and lower are the trees derived from analyses of nonsynonymous (*d*_*N*_) and synonymous (*d*_*S*_) substitutions, respectively. MAGGs, TSTGs, and “all genes” are represented (from left to right). The *d*_*N*_*/d*_*S*_ for each species is given in parentheses

Consensus evolutionary trees were generated both for *d*_N_ and *d*_S_ substitutions in MAGGs, TSTGs and “all genes” based on the unrooted tree estimated in our previous phylogenetic analysis [30]. The rates of *d*_N_ and *d*_S_ were estimated over all branches. MAGGs showed significantly higher *d*_N_ relative to TSTGs and to “all genes,” in all the six *Glossina* lineages analyzed (Tukey HSD test, *P* < 0.05) (Fig. 4; Table S2). But the level of selective pressure on MAGGs is heterogeneous both within and among the species groups. Within the savannah *Morsitans* group, the *G. austeni* lineage displays the highest *d*_N_/*d*_S_ value (0.293), while in the riverine *Palpalis* group, *G. fuscipes* shows the highest estimate (*d*_N_/*d*_S_ = 0.307).

For TSTGs, the level of selective pressure in the riverine *Palpalis* group appears to be higher with respect to the *Morsitans* species (Fig. 4). It is noteworthy that the differences in selective pressure on TSTGs among the species are maintained also at the level of “all genes” category (Fig. 4).

### Candidates for selection in *Glossina* lineages include genes encoding ejaculate proteins

**Fig. 5:**
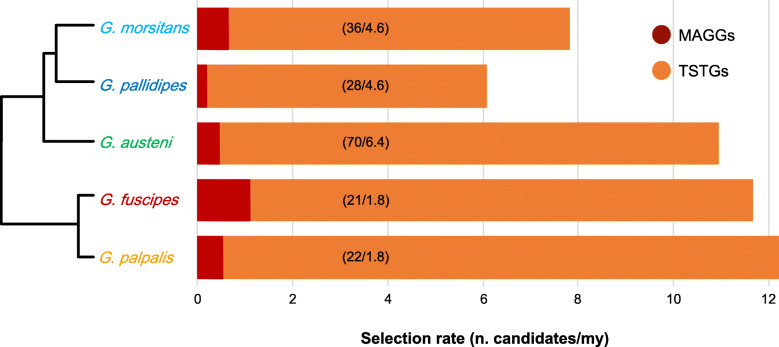
Selection rate in the different lineages. Rate of positively selected genes (*n* candidates/mya): bars are number of candidate genes (identified using branch and/or branch-site codon substitution model tests in PAML) per million years (based on time tree) in each of the *Glossina* terminal branches

Different models of substitution rates across coding sites (i.e., site, branch, and branch-site tests) allowed the identification of 750 genes as candidates for selection in at least one test in at least one branch of the phylogeny of the *Morsitans* and *Palpalis* groups (i.e., *G. m. morsitans*, *G. pallidipes*, *G. austeni*; *G. palpalis*, *G. fuscipes*) using a false discovery rate of 20% as a threshold. Among these, 176 were from testes and 10 from MAGs (Table S4, S5). Globally, when the pattern of evolution of the MAGGs and TSTGs is evaluated in relation to the divergence time of the species, it appears that the selection rate is higher in the *Palpalis* than in the *Morsitans* group, with the exception of *G. austeni* (Fig. 5).

**Fig. 6:**
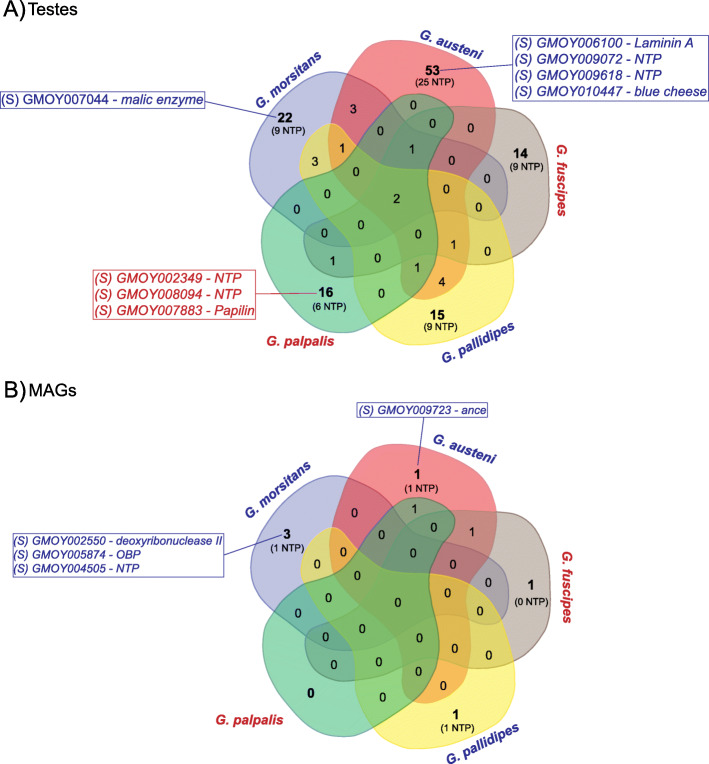
Reproductive genes under selective pressure in the *Glossina* lineages. Numbers of unique and shared candidates for TSTGs (**A**) and MAGGs (**B**) are reported. Among these groups, genes found to encode spermatophore proteins in *G. m. morsitans* (29) are indicated in boxes and labelled with an (S). In brackets, the novel tsetse protein-coding genes (NTP) are indicated. Species belonging to the *Morsitans* and *Palpalis* species group are indicated in blue and red, respectively

The testes and MAG candidates for selection are differently distributed among the species (Fig. 6; Tables S4, S5). These genes include sequences which, in *G. m. morsitans*, encode for ejaculate proteins (i.e., 15 from testes and 7 from MAGs), and many novel tsetse protein (NTP) genes (i.e., 83 from testes and 4 from MAGs).

As far as it concerns testes genes, the *Morsitans* species group globally displays a higher number of candidates for selection than *Palpalis* (106 vs 36) (Fig. 6; Table S4). Indeed, *G. austeni* has 53 candidates, with 43% of them (*n* = 23) being NTPs. Four of these genes encode for predicted ejaculate proteins: *Laminin A* (GMOY006100), *blue cheese* (GMOY010447), and two NTP genes (GMOY009072 and GMOY009618). In *G. m. morsitans*, out of the 22 candidates, 40% (*n* = 9) are NTP genes and include the ejaculate protein gene *Malic enzyme* (GMOY007044) (Fig. 6; Table S4). In the more recently evolved *Palpalis* group, *G. palpalis* has 16 candidates for selection, with 37% being NTP genes, and including three predicted ejaculate protein genes: two NTP genes (GMOY002349, GMOY008094) and the extracellular matrix proteoglycan-like sulfate glycoprotein *Papilin* (GMOY007883) [44]. In *G. fuscipes*, among the 14 candidates, 64% (*n* = 9) are NTP genes (Fig. 6; Table S4).

As far as it concerns the MAG candidates for selection, the three genes in *G. m. morsitans* encode for ejaculate proteins [29], i.e., *deoxyribonuclease-2-alpha* (GMOY002550), a putative OBP (GMOY005874), and the NTP gene GMOY004505 (Fig. 6; Table S5). In *G. austeni* and in *G. pallidipes*, only one candidate was identified: the ejaculate protein-coding gene *ance* (GMOY009723), and one NTP gene (GMOY002583), respectively. In the *Palpalis* species group, only one candidate for selection was identified, the *transmembrane channel 1* (GMOY005914) in *G. fuscipes*.

## Discussion

Using a comparative genomic approach, we identified and screened for signals of selection in the male reproductive genes of six *Glossina* species representative of the *Fusca*, *Morsitans*, and *Palpalis* sub-genera. The data indicate that the overall evolutionary patterns of male reproductive genes are consistent with the time-calibrated phylogeny of these *Glossina* groups. However, the evolutionary rate of genes from the MAGs is faster than that of testes-specific/enriched genes and is heterogeneous among and within the species groups. Genes encoding ejaculate proteins and novel tsetse proteins (NTPs) have been found to be under selective pressure.

### *Glossina* male reproductive genes’ evolution and viviparity

A large dataset of male reproductive genes remained conserved over the long evolutionary time scale (estimated at 26.7 mya) across the genome of the six *Glossina* species from *Fusca*, *Morsitans*, and *Palpalis* sub-genera (from 1704 genes in *G. brevipalpis* / *Fusca* group, to 2016 genes in *G. m. morsitans / Morsitans* group). This male reproductive gene conservation may be interpreted as the consequence of the strong functional selective pressure on the male imposed by the viviparous reproductive condition (see [45] for a review). It is noteworthy that more than half of these conserved sequences are NTP genes that are unique to the *Glossina* genus and are candidates for selection in the different lineages [12, 46].

Given that viviparity in *Glossina* leads to the production of only a few progeny [24], the conflict between competing individual males to increase their success for fertilization is exacerbated with respect to oviparous species [45, 47]. But also the female exerts pressure on the male through post-mating sexual selection by biasing male fertilization success through cryptic female choice and sperm competition [36, 48, 49]. An additional role in these post-mating conflicts is played by the male ejaculate transferred to the female and the female receptivity [50–52]. As in most animals, the ejaculate of *G. m. morsitans* is derived from the male reproductive organs, testes and MAGs, with each tissue making specific and distinct contributions to the ejaculate composition. The testes express genes primarily associated with spermatogenesis (and its regulation), sperm storage, transfer, and fertilization functions. Male accessory glands express a smaller set of genes at a high level that encode for the abundant non-sperm associated components of the ejaculate [29]. It is noteworthy that 72% of the testes and MAG sequences are conserved across the species analyzed. These genes probably serve different functions and are under different and contrasting intensities of selective pressure. Indeed, TSTGs display, across all functional GO categories, an average *d*_N_/*d*_S_ estimate of 0.1, suggesting the action of strong and pervasive purifying selection. By contrast, MAGGs, though less numerous, display an average *d*_N_/*d*_S_ estimate of 0.2, but they are heterogeneous for evolutionary rates. This finding indicates the presence of a combination of purifying, positive, and relaxed selection across the GO categories, in agreement with their different functional roles in post-mating female responses and male reproductive success as found in other insects [53, 54] and suggested for *G. m. morsitans* [29]. Novel tsetse genes, genes with predicted odorant binding, enzyme inhibitor, and protein binding functions are the categories that are under higher selective pressure in the MAGs with respect to the testes. These gene classes code for the most abundant proteins in the *G. m. morsitans* ejaculate [29]. The presence of OBP genes that are under selective pressure in the MAGs that code for proteins transferred to the female in the ejaculate raises the interest in their function in *Glossina* reproductive biology. Recent data from *Drosophila*, mosquitoes, and fruit flies suggest that OBPs may be involved in bringing odorants or pheromones next to the odorant receptors present in the female reproductive tracts or carry male-specific molecules into female tissues to elicit behavioral responses [55–62]. Whether tsetse OBP genes expressed in the male reproductive tract have similar functions and influence fertility and fecundity are open questions.

We previously explored the level of conservation of *Glossina* male reproductive genes in oviparous insects such as *Musca domestica*, *Drosophila melanogaster*, *Aedes* spp., and *Anopheles gambiae* that also display different strategies for ejaculate delivery and assembly [29]. Apart from the NTP genes, which are specific to *Glossina*, the conservation of tsetse male reproductive genes followed the expected phylogenetic relationships among the considered taxa, with sperm-related genes being more conserved than seminal fluid genes [29]. This trend is particularly evident when comparing *G. m. morsitans* with *An. gambiae*, as seminal fluid components of the mosquito mating plug did not display any similarity to those of the tsetse spermatophore. Considering the evolutionary rates of male reproductive genes in other insects, the conserved MAG genes within the *An. gambiae sensu lato* species complex that include those encoding plug components, are subjected to a relaxation of purifying selection [7], as we observed in *Glossina*.

### The evolution of male reproductive genes in the different *Glossina* lineages may be affected by habitat restrictions and behavior

The consensus evolutionary trees, generated across the lineages both for the *d*_N_ and *d*_S_ substitutions in MAGGs, TSTGs, and “all genes” (Fig. 4), reflect the long and divergent demographic history of the *Fusca*, *Morsitans*, and *Palpalis* groups. Indeed species from these sub-genera display an allopatric distribution, testifying their long separation during which random drift, fluctuating climates, and different selective regimes have promoted divergence and defined restricted habitat specificity for each group and species [30]. *Fusca*, a sister group to other tsetse lineages [63], contains species, such as *G. brevipalpis*, that occupy the ancestral forest habitat [64], and it was the first lineage to differentiate at 26.7 mya. This finding is consistent with the Oligocene *Glossina* fossil records found in Colorado (USA), which are dated from 33.9 to 23 mya [65, 66]. The *Morsitans* group diverged from *Fusca* at a mean of 10.8 mya, with most posterior estimates between 14.8 and 7.2 mya. These estimates are compatible with a *Morsitans* adaptation to the savannah habitat that appeared in sub-Saharan Africa about 7–8 mya (Miocene to Pliocene boundary) [67, 68]. Similarly, the split of the *Palpalis* group from *Morsitans* may have been an adaptation to riverine habitats that occurred at about 10.8 mya. The riverine group currently inhabits the vegetation close to water sources [69]. The habitat restrictions for these species had an important effect on their behavior, leading to specializations in inter- and intra-sexual interactions, to ensure efficient insemination [70, 71]. In this context, the male reproductive genes likely played a key role.

We found that the level of selective pressure on male reproductive genes, especially in the MAGs, is higher in the *Palpalis*/riverine species than in the *Morsitans*/savannah group. This trend is particularly evident in *G. fuscipes*. *Palpalis* species, living in narrow riverine habitats, suffer seasonal demographic fluctuations. During the dry season, populations undergo demographic contractions with the remaining flies concentrating in moist refugia. At the end of the dry season, within the residual population emerging after the bottleneck, the strength of male competition increases because of the greater number of interactions for achieving copulation. In these expanding populations, higher remating rates have been observed than in the population contractions typical of the dry season [36]. Such remating rates are not unexpected given that cryptic female choice and multiple mating may provide a buffer against changing eco-climates [72].

By contrast, *Morsitans* species occupy a more stable environment [34]. They inhabit extensive, relatively homogeneous, and open woodlands, and their populations are strongly allopatric with restricted gene flow and a relatively high dispersal capacity. Remating rate is lower [73], suggesting the presence of a less intense sexual selective pressure. Among these species, *G. austeni* is an exception both in terms of habitat restriction and of candidate male reproductive genes under selection. Indeed, this species is confined to a narrow discontinuous belt on the East African coast and it does not move far from its breeding habitats [74]. Remating rates in this species are high [73, 75, 76]. By contrast with the other members of the *Morsitans* group, the males display a very precocious MAG and spermatogenesis machinery development that permits the production of the ejaculate for an efficient insemination within the first 24 h after eclosion [75, 76]. This behavior may support the high selective pressure we found on MAGGs and the higher number of testes candidate genes for selection with respect to the other species.

## Conclusions

Here we attempted to interpret the evolution of male reproductive genes in *Glossina* species considering their peculiar adaptations, such as obligate viviparity and strict habitat specificity. With respect to viviparity, the observed signatures of gene evolution may be considered in the light of the very tight co-evolution between male and female genomes. Indeed, viviparity creates a post-fertilization arena for genomic conflicts that are absent in ovipositing species [45, 47, 77–80]. These conflicts can arise between the mother and the developing embryo in the uterus, but also between the maternal and paternal genomes within the embryo (see [45] for a review). As a consequence, a continuous antagonistic co-evolution occurs between the parental genomes, thus accelerating inter-population post-zygotic isolation and, ultimately, speciation. Ecological restrictions that affect reproductive behavior of these species may further shape such antagonistic co-evolution. *Glossina* flies offer an ideal opportunity to investigate these patterns and the underlying regulatory mechanisms. These aspects are of great interest considering the important role these flies play as vectors of parasitic trypanosomes in Africa. In the absence of vaccines, the best available method to limit the burden of disease is to control *Glossina* as vectors. Unfortunately, most vector control strategies rely on the use of insecticides. Given the strong limitations imposed by viviparity on reproductive output, development of effective strategies to interfere with *Glossina* reproduction in the field is a desirable approach. Our list of conserved NTP genes is a valuable starting point for the selection and testing of new targets for the identification of mechanisms regulating fertility in these species. This is a necessary step towards the development of translational applications.

## Methods

### Time-calibrated phylogeny

Data from *G. m. morsitans* genome were used to conduct a comprehensive multi-locus dated phylogenetic analysis in the context of the available genome data from *Glossina* species, i.e., *G. pallidipes*, *G. austeni*, *G. fuscipes*, *G. palpalis*, and *G. brevipalpis* [30, 39]. To maximize the power of our phylogenetic analysis, several outgroup species were incorporated into the study. These include *Musca domestica* as a representative of the Calyptrates, *Drosophila melanogaster*, *D. ananassae*, and *D. grimshawi* representing the Acalyptrates, and *Lutzomyia longipalpis* and *Anopheles gambiae* representing the Nematocera. The strains and the genome resources are given in Table S6.

Orthologous genes across the six *Glossina* and the outgroup species were identified following the same procedure as described by Attardo and colleagues [30] using *G. m. morsitans* as focal species, aligning individually using MAFFT [81] and concatenating in a super-alignment of 478,617 nucleotide positions. We inferred divergence estimates using PhyloBayes (4.1; https://megasun.bch.umontreal.ca/People/lartillot/www/download.html) [82] on the gblocked amino acid dataset. Divergence rates were calibrated at four nodes: (a) a minimum of 33 million years (my) for the divergence of the *Glossina* genus based on the oldest *Glossina* fossil from the Florrisant Beds estimated to be late Eocene [83]; (b) a maximum of 294.5 and a minimum of 238.5 my for the *Drosophila*/*Anopheles* split [84]; (c) a minimum of 64 my for the *Glossina*/*D. melanogaster* split; (d) a minimum of 44 my for the *Drosophila* genus divergence [85]. All node constraints were treated as soft allowing 5% of mass allocation outside both boundaries [86]. A LogNormal relaxed molecular clock, a GTR + G replacement model, and a Birth and Death tree prior were used. Two independent chains were run until consensus trees converged on extremely similar divergence estimates. Final divergences were calculated on one of the chains after exclusion of the first 10% of sampled trees as burn-in.

### Estimates of divergence and test for positive selection of male reproductive genes

#### Orthologous gene set identification

The coding sequences of all six species were obtained from VectorBase (www.vectorbase.org), and then used in pairwise BLASTn between the *G. m. morsitans* sequences and those of the other *Glossina* species. To identify orthologs, we used a reciprocal-best-BLAST-hits (RBH) approach [87] in which *G. m. morsitans* was used as focal species. We retrieved a total of 8088 orthologous groups represented by at least five *Glossina* species (*G. morsitans* being always one of the five), 5513 of which had all six orthologous sequences. We then partitioned our dataset based on a stringently selected set of genes which, in *G. m. morsitans*, displayed enriched transcription in male accessory glands (MAGGs) or testes (TSTGs), respectively (≥ 5-fold) [29].

#### Analyses of the rate of evolution of male reproductive genes in *Glossina* species

All orthologous sequences were aligned using PRANK (version 14.06.03) in codon mode [88], as implemented in TranslatorX [89], which aligns protein-coding nucleotide sequences based on their corresponding amino acid translations. To minimize the possibility of spurious matches, orthologous sets with sequences shorter than 50 amino acids were removed. We also used a custom perl script to remove problematic alignment regions using an approach similar to that proposed by Han and colleagues and Ramasamy and colleagues [90, 91]. Rates of molecular evolution were determined for *Glossina* orthologs using PAML 4.7 [92] based on the unrooted tree estimated in our phylogenetic analysis [30] (see below), which has topology *(((G. m. morsitans, G. pallidipes), G. austeni), (G. fuscipes, G. palpalis), G. brevipalpis)*. The rate of nonsynonymous substitution, *d*_N_ (leading to amino acid changes), and synonymous substitution, *d*_S_ (which should accumulate neutrally), were estimated over all branches of the phylogenetic tree using the “free-ratio” model (M0 [93]; model = 1 and NSsites = 0). This model allows ω = *d*_N_/*d*_S_, i.e., the level of selective pressure experienced by a gene, to vary among branches of the tree.

We then used PAML to test different models of substitution rates across coding sites [94, 95], with the aim of detecting genes that either evolved at a different rate or that underwent positive selection along one of the *Glossina* lineages. To maximize statistical power, these tests were performed only on orthologous sets containing at least five species. In the first test, we compared models that assumed one or more substitution rates across the phylogeny. The first of such models is the basic “one-ratio” branch model (M0), which assumes a constant *ω* across the phylogeny (model = 0 and NSsites = 0). Following the manual recommendations, this model was used to obtain the branch lengths for each gene tree, which were then copied into the tree structure file to be used with the branch and site substitution models. The likelihood of the M0 model was compared to that of a branch model that assumed two *ω* values. In the branch model, one *ω* value represents the *Glossina* species with the exception of *G. brevipalpis* (the so-called foreground branch), and one *ω* value for the rest of the tree (the background branches; model = 2 and NSsites = 0). *Glossina brevipalpis* is not included with the rest of the *Glossina* species as using an unrooted tree we cannot separate the processes that acted along its lineage from those that took place in the lineage subtending the clade containing the other five species. Subsequently, the value of twice the difference between the two likelihoods was tested using a *χ*^2^ test with 1 degree of freedom. The occurrence of positive selection was tested by the branch-site test. In this test (branch-site model A, test 2 [96]), *ω* can vary both among sites in the protein and across branches on the tree (model = 2, NSsites = 2). As for the branch model, we used tree structures with branch lengths estimated by model M0. The null model fixed *ω*_2_ = 1 (fix_omega = 1, omega = 1), whereas the positive selection model allowed *ω*_2_ > 1 (fix_omega = 0, omega = 1). The likelihood ratio test had 1 degree of freedom. The occurrence of positive selection was also tested by comparing (nearly) neutral models to models that allow for the occurrence of positive selection (site tests). In a first approach, we compared the likelihood of a model (M1a; model = 0 and NSsites = 1) that assumes two sets of sites with neutral (*ω* = 1) or nearly neutral evolution (0 < *ω* < 1), to a model with an additional class of sites with *ω* > 1 (M2a; model = 0 and NSsites = 2). In a second more realistic approach, we compared the likelihood of a model where ten site classes have *ω* values drawn from a *β* distribution (M7; model = 0 and NSsites = 7) to a model that incorporates an additional class of sites under positive selection (M8; model = 0 and NSsites = 8). In these cases, each comparison was tested using a *χ*^2^ test with 2 degrees of freedom. To account for multiple testing, we also estimated the false discovery rate (FDR) of each test using the *q*-value approach [97] implemented in R [98]. Genes were defined as being under significant positive selection if they had an associated *q*-value < 0.20. We note that these analyses are conservative regarding the frequency of positive selection, since the reciprocal-best-hit approach is prone to miss genes with high sequence divergence, including those that underwent particularly intense divergent adaptive evolution.

The intersections among the MAGGs and TSTGs found to be under selection in the different *Glossina* lineages were visualized by a Venn diagram (http://bioinformatics.psb.ugent.be/webtools/Venn/).

#### Functional classification of *Glossina* male reproductive sequences

Functional classification of tsetse orthologs was performed using Argot^2^ [99] and the Blast2GO software v.2.8 (https://www.blast2go.com/b2ghome). The CDS of *G. m. morsitans* reproductive genes were used to perform BLASTx against the NCBI non-redundant (nr) database (*e*-value < 10^− 10^). For Gene Ontology mapping (GO; http://www.geneontology.org) we used Blast2GO to extract GO terms associated with homologies identified by NCBI’s BLAST. We retained annotations with *e*-value < 10^− 10^. We then performed InterPro and InterProScan [100] searches remotely from Blast2GO via the InterPro EBI web server and merged InterProScan GOs with the original GO annotations. The notched box plot figures showing the *d*_N_/*d*_S_ relative to each GO functional class (Molecular Function Level III) were developed using R Studio [101]. In the case of novel tsetse proteins (NTP), i.e., sequences sharing no similarity to sequences present in the GenBank database [29] and for which GO terms could not be assigned, but displaying high *d*_*N*_*/d*_*S*_ values, potential functional roles have been inferred, when possible, using the InterPro results.

## Supplementary Information

**Additional file 1: Table S1.** Level of selective pressure across the 8088 genes with orthologs in at least five *Glossina* species. Selective pressure (*d*_*N*_*/d*_*S*_) values were estimated as fixed in the whole phylogeny. Genes over-expressed in MAGs or testes are indicated.

**Additional file 2: Table S2.** Level of selective pressure across the 5513 genes with orthologs in all six *Glossina* species. Selective pressure (*d*_*N*_*/d*_*S*_) values were estimated for each branch of the phylogeny. Genes over-expressed in MAGs or testes are indicated.

**Additional file 3: Table S3.** List of TSTGs and MAGGs and their distribution among Gene Ontology functional categories. The presence of their protein products in *G. m. morsitans* spermatophore are reported. Omegafix values are indicated for each gene.

**Additional file 4: Table S4.** Testes genes (*n* = 176) in *G. m. morsitans, G. austeni*, *G*. *fuscipes*, *G. pallidipes* and *G. palpalis* tested for positive selection after site (A and B), branch (Br) and branch-site (BrS) models. Dataset column indicates the number of species in which ortholog sequences of each *G. m. morsitans* gene were identified (aus = *G. austeni*; b = *G. brevipalpis*; f = *G. fuscipes*; pai = *G. pallidipes*; ppi = *G. palpalis*). Gene ID column reports *G. m. morsitans* orthologs. NTP indicate genes encoding Novel Tsetse Proteins. * FDR < 0.20; ** FDR < 0.05; *** FDR < 0.005.

**Additional file 5: Table S5.** Number of MAG genes (*n* = 10) in *G. m. morsitans, G. austeni*, *G*. *fuscipes*, *G. pallidipes* and *G. palpalis* tested for positive selection after site (A and B), branch (Br) and branch-site (BrS) models. Dataset column indicates the number of species in which ortholog sequences of each *G. m. morsitans* gene were identified (b = *G. brevipalpis*; ppi = *G. palpalis*). Gene ID column reports *G. m. morsitans* orthologs. NTP indicate genes encoding Novel Tsetse Proteins. * FDR < 0.20; ** FDR < 0.05; *** FDR < 0.005.

**Additional file 6: Table S6.** List of species, strains and genome resources used in this study.

## Data Availability

The data that support the findings of this study are available in the supplementary material of this article. The pipeline used for the identification of the orthologous gene set and the dataset used in PhyloBayes are available in the Open Science Framework data repository at https://osf.io/zjbdx/?view_only=1b0935ab0e5a447ab3b68dad1aa5a3bd [102].
