# Supplementary material for: Viviparity and habitat restrictions may influence the evolution of male reproductive genes in tsetse fly (Glossina) species
Source: BMC Biol. 2021 Sep 23;19:211. doi: 10.1186/s12915-021-01148-4 (PMC8461966; doi:10.1186/s12915-021-01148-4)
Supplement: Supplementary file 4 — Additional file 4: Table S4. Testes genes (n = 176) in G. m. morsitans, G. austeni, G. fuscipes, G. pallidipes and G. palpalis tested for positive selection after site (A and B), branch (Br) and branch-site (BrS) models. Dataset column indicates the number of species in which ortholog sequences of each G. m. morsitans gene were identified (aus = G. austeni; b = G. brevipalpis; f = G. fuscipes; pai = G. pallidipes; ppi = G. palpalis). Gene ID column reports G. m. morsitans orthologs. NTP indicate genes encoding Novel Tsetse Proteins. * FDR < 0.20; ** FDR < 0.05; *** FDR < 0.005. [file 12915_2021_1148_MOESM4_ESM.docx]

**Supplementary Table 4. Testes genes (*n* = 176) in *G. m. morsitans, G. austeni*, *G*. *fuscipes*, *G. pallidipes* and *G. palpalis* tested for positive selection after site (A and B), branch (Br) and branch-site (BrS) models.** Dataset column indicates the number of species in which ortholog sequences of each *G. m. morsitans* gene were identified (aus=*G. austeni*; b=*G. brevipalpis*; f= *G. fuscipes*; pai=*G. pallidipes*; ppi=*G. palpalis*). Gene ID column reports *G. m. morsitans* orthologs. NTP indicate genes encoding Novel Tsetse Proteins. * FDR < 0.20; ** FDR < 0.05; *** FDR < 0.005. ^§^Genes encoding spermatophore proteins in *G. m. morsitans* (Scolari et al., 2016).

|  |  |  | **Site test** | | ***G.morsitans*** | | ***G.austeni*** | | ***G.fuscipes*** | | ***G.pallidipes*** | | ***G.palpalis*** | |
| --- | --- | --- | --- | --- | --- | --- | --- | --- | --- | --- | --- | --- | --- | --- |
| **Dataset** | **Gene ID** | **Putative function** | **A** | **B** | **Br** | **BrS** | **Br** | **BrS** | **Br** | **BrS** | **Br** | **BrS** | **Br** | **BrS** |
| all | GMOY000082 | NTP |  |  |  |  | * |  |  |  |  |  |  |  |
| all | GMOY000168 | snazarus | ** | ** |  |  |  | * |  |  |  |  |  |  |
| all | GMOY000485 | NTP |  |  |  |  |  | * |  |  |  |  |  |  |
| all | GMOY000539 | ataxin-2 |  |  |  |  | * |  |  |  |  |  |  |  |
| all | GMOY000654 | Palmitoyltransferase |  |  | * |  |  |  |  |  |  |  |  |  |
| all | GMOY000863 | NTP |  | * |  |  |  |  |  |  |  | ** |  |  |
| all | GMOY001139 | outer segment 1 |  |  |  |  | * |  |  |  |  |  |  |  |
| all | GMOY001316 | Serine/threonine-protein phosphatase 2A 55 kDa regulatory subunit B |  |  |  |  |  |  |  |  | * |  |  |  |
| all | GMOY001320 | NTP |  |  |  |  |  |  |  |  |  | * |  |  |
| all | GMOY001350 | easily shocked | ** | *** |  |  |  |  |  |  |  |  |  |  |
| all | GMOY001744 | NTP |  |  |  |  |  |  | * |  |  |  |  |  |
| all | GMOY001748 | NTP |  |  | * |  |  |  |  |  |  |  |  |  |
| all | GMOY001860 | kismet |  |  |  |  |  |  |  |  |  |  |  | ** |
| all | GMOY001887 | lambik |  |  |  | ** |  |  |  |  |  |  |  |  |
| all | GMOY001913 | NTP |  |  |  |  | * |  |  |  |  |  |  |  |
| all | GMOY001941 | endocytosis/signaling protein EHD1 |  |  |  |  |  |  |  |  |  |  | * |  |
| all | GMOY001977 | NTP |  |  |  |  | * |  |  |  |  |  |  |  |
| all | GMOY002025 | tyrosine-protein kinase |  |  | * |  |  |  |  |  |  |  |  |  |
| all | GMOY002064 | optix-binding protein |  | * |  |  |  |  |  |  |  |  |  |  |
| all | GMOY002287 | acinus | * | ** |  |  |  |  |  |  |  |  |  |  |
| all | GMOY002349^§^ | NTP |  |  |  |  |  |  |  |  |  |  | ** | *** |
| all | GMOY002375 | short spindle |  |  | ** |  |  |  |  |  |  |  |  |  |
| all | GMOY002564 | NTP |  |  |  |  |  |  | * |  |  |  |  |  |
| all | GMOY002800 | furin |  | *** |  |  |  |  |  |  |  |  |  |  |
| all | GMOY002820 | rabconnectin-3A |  |  |  |  | * |  |  |  |  |  |  |  |
| all | GMOY002841 | NTP |  |  |  |  |  |  |  |  |  |  | *** |  |
| all | GMOY002881 | NTP |  | * |  |  |  |  |  |  |  |  |  |  |
| all | GMOY003057 | ecdysone-induced protein 63E | *** | *** |  |  |  |  |  |  |  |  |  |  |
| all | GMOY003081 | mitogen-activated protein kinase organizer 1 |  |  |  | * |  |  |  |  |  |  |  |  |
| all | GMOY003155 | NTP |  |  |  |  | * |  |  |  |  |  |  |  |
| all | GMOY003188 | meiotic recombination |  |  |  |  |  |  |  |  |  |  | ** |  |
| all | GMOY003237 | Cd GTPase activating protein-related |  |  |  |  | ** |  |  |  |  |  |  |  |
| all | GMOY003370 | Tyrosine-protein kinase | ** | ** |  |  |  |  |  |  |  |  | * |  |
| all | GMOY003432 | NTP |  |  |  |  | * |  |  |  |  |  |  |  |
| all | GMOY003582 | NTP |  |  |  |  | * |  |  |  |  |  |  |  |
| all | GMOY003628 | katanin p60 ATPase-containing subunit A1 | *** | *** |  |  |  |  |  |  |  |  |  |  |
| all | GMOY003636 | NTP |  | ** |  |  |  |  |  |  |  |  |  |  |
| all | GMOY003638 | NTP |  |  |  |  | * |  |  |  |  |  |  |  |
| all | GMOY003771 | NTP |  |  |  |  | * |  |  |  |  |  |  |  |
| all | GMOY003823 | NTP |  |  |  |  |  | * |  |  |  |  |  |  |
| all | GMOY003915 | cullin-3 | *** | *** | *** |  | *** |  | *** |  | *** |  | *** |  |
| all | GMOY004004 | NTP | *** | *** |  |  |  |  |  |  |  |  |  |  |
| all | GMOY004064 | DNA repair protein REV1 |  |  |  |  |  |  |  | ** |  |  |  |  |
| all | GMOY004222 | Ionotropic glutamate receptor |  |  |  | ** |  |  |  |  |  |  |  |  |
| all | GMOY004238 | NTP |  | * |  |  |  |  |  |  |  |  |  |  |
| all | GMOY004338 | NTP |  |  |  |  | * |  |  |  |  |  |  |  |
| all | GMOY004441 | NTP | * | *** |  |  |  |  |  |  |  |  |  |  |
| all | GMOY004688 | NTP |  |  | * |  |  |  |  |  |  |  |  |  |
| all | GMOY004744^§^ | alpha actinin | *** | *** |  |  |  |  |  |  |  |  |  |  |
| all | GMOY004783 | phosphofructokinase |  |  |  |  | * |  |  |  | *** |  |  |  |
| all | GMOY004875 | phosphoinositide phospholipase C | * | ** |  |  |  |  |  |  |  |  |  |  |
| all | GMOY004932 | GTPase activating protein and centrosome-associated |  | * |  |  |  |  |  |  |  |  |  |  |
| all | GMOY005070 | dynein heavy chain 64C |  |  |  |  | * |  |  |  |  |  |  |  |
| all | GMOY005075 | NTP |  |  |  |  | *** |  |  |  |  |  |  |  |
| all | GMOY005314 | DNA fragmentation factor-related protein | * | *** |  |  |  |  |  |  |  |  |  |  |
| all | GMOY005361 | NTP |  |  |  |  |  |  |  |  | * |  |  |  |
| all | GMOY005379 | megalin |  |  |  |  | *** |  |  |  |  |  |  |  |
| all | GMOY005409 | component of oligomeric golgi complex |  |  |  |  |  |  |  |  |  |  | * |  |
| all | GMOY005561 | huvenile hormone epoxide hydrolase |  |  |  |  | ** |  |  |  |  |  |  |  |
| all | GMOY005627 | monensin sensitivity | ** | *** | ** |  | ** |  |  |  |  |  |  |  |
| all | GMOY005703^§^ | myosin heavy chain | *** | *** |  |  |  |  |  |  |  |  |  |  |
| all | GMOY005863 | salivary mucin | *** | *** |  |  |  |  |  |  |  |  | ** |  |
| all | GMOY005903 | netrin-B |  |  |  |  |  |  |  |  |  | * |  |  |
| all | GMOY005954 | NTP | *** | *** |  | *** |  |  |  |  |  |  |  |  |
| all | GMOY006023 | kelch |  |  |  |  | * |  |  |  |  |  |  |  |
| all | GMOY006100 | laminin A |  |  |  |  | * |  |  |  |  |  |  |  |
| all | GMOY006119 | Leucine zipper and EF-hand containing transmembrane protein |  |  | * |  |  |  |  |  |  |  |  |  |
| all | GMOY006132 | NTP |  | * |  |  |  |  |  |  |  |  |  |  |
| all | GMOY006227 | yolk protein 3 | *** | *** |  |  |  |  |  |  |  |  |  |  |
| all | GMOY006302 | tetraspanin |  |  |  |  |  |  | ** | ** |  |  |  |  |
| all | GMOY006377 | nejire |  |  |  |  | * |  |  |  |  |  |  |  |
| all | GMOY006415 | NTP |  |  | * |  |  |  |  |  |  |  |  |  |
| all | GMOY006503 | beta-hexosaminidase |  |  |  |  | ** |  |  |  |  |  |  |  |
| all | GMOY006579 | DNA-directed RNA polymerase subunit |  | ** |  |  | * |  |  |  |  |  |  |  |
| all | GMOY007021 | NTP |  | ** |  |  |  |  |  |  |  |  |  |  |
| all | GMOY007044^§^ | malic enzyme |  |  |  | * |  |  |  |  |  |  |  |  |
| all | GMOY007157 | NTP |  | * |  |  |  |  |  |  |  |  |  |  |
| all | GMOY007253 | ryanodine receptor | *** | *** |  |  |  |  |  |  | *** | *** |  |  |
| all | GMOY007388 | enhancer of decapping |  |  |  |  | ** |  |  |  |  |  |  |  |
| all | GMOY007547 | monocarboxylate transporter 8 | *** | *** |  |  | ** |  | *** | *** | *** | ** |  |  |
| all | GMOY007750 | gamma-tubulin complex component | * | * |  |  |  |  |  |  |  |  |  |  |
| all | GMOY007797 | NTP |  |  |  |  | * |  |  |  |  |  |  |  |
| all | GMOY007818 | NTP |  |  |  | ** |  |  |  |  |  |  |  |  |
| all | GMOY007838 | NTP |  | * |  |  |  |  |  |  |  |  |  |  |
| all | GMOY007883 | papilin |  |  |  |  |  |  |  |  |  |  |  | * |
| all | GMOY008094 | NTP |  |  |  |  |  |  |  |  |  |  |  | ** |
| all | GMOY008119 | zizimin-related |  |  |  |  |  |  |  |  |  | * |  |  |
| all | GMOY008138 | kurz |  |  |  |  | * |  |  |  |  | * |  |  |
| all | GMOY008388 | Disco-related |  |  |  | * |  |  |  |  |  |  |  |  |
| all | GMOY008433 | NTP |  |  |  |  |  | * |  |  |  |  |  |  |
| all | GMOY008456 | ADAM metallopeptidase with thrombospondin type 1 motif A |  |  |  |  |  | * |  |  |  |  |  |  |
| all | GMOY008545^§^ | NTP |  | * |  |  | ** |  |  |  |  |  |  |  |
| all | GMOY008639 | NTP | ** | *** |  |  |  |  | * |  |  |  |  |  |
| all | GMOY008690 | dilute class unconventional myosin |  |  |  |  |  | *** |  |  |  |  |  |  |
| all | GMOY008811 | lethal (2) giant discs |  |  |  |  | * |  |  |  |  |  |  |  |
| all | GMOY008814 | cation-transporting ATPase |  |  |  |  | ** |  |  |  |  |  |  |  |
| all | GMOY008876 | TBP-associated factor |  | * |  |  |  |  |  |  |  |  |  |  |
| all | GMOY008882 | molecule interacting with CasL |  |  |  |  | * |  |  |  |  |  |  |  |
| all | GMOY009072 | NTP |  |  |  |  | * |  |  |  |  |  |  |  |
| all | GMOY009123 | NTP |  |  |  |  | * |  |  |  |  |  |  |  |
| all | GMOY009167 | NTP |  |  |  |  |  |  |  |  |  |  | *** |  |
| all | GMOY009235 | histone acetyltransferase |  |  |  |  |  |  |  |  |  |  |  | *** |
| all | GMOY009251 | NTP |  |  |  |  |  |  |  |  |  | * |  |  |
| all | GMOY009256 | NTP |  |  | ** |  | * |  |  |  |  |  |  |  |
| all | GMOY009618^§^ | NTP |  | * |  |  | *** | * |  |  |  |  |  |  |
| all | GMOY009682 | cell division cycle 7-related kinase |  |  |  |  | *** |  |  |  |  |  |  |  |
| all | GMOY009683 | profilin |  |  |  | *** |  |  |  |  |  | ** |  |  |
| all | GMOY009744^§^ | NTP | * | * |  |  |  |  |  |  |  |  |  |  |
| all | GMOY009833 | embryonic lethal abnormal vision |  |  |  |  |  |  | ** |  |  |  |  |  |
| all | GMOY009862 | BIR repeat containing ubiquitin-conjugating enzyme |  | * |  | * | *** |  |  |  |  |  |  |  |
| all | GMOY009931 | Lk6 kinase |  |  |  |  |  | ** |  |  |  |  |  |  |
| all | GMOY009936 | NTP |  |  |  |  |  | ** |  |  |  |  |  |  |
| all | GMOY009959 | Rho GTPase activating protein at 93B |  |  |  |  |  |  |  | * |  |  |  |  |
| all | GMOY010088 | NTP |  |  |  |  | * |  |  |  |  |  |  |  |
| all | GMOY010094 | Daxx-like protein |  | * |  |  |  |  |  |  |  |  |  |  |
| all | GMOY010114 | rhomboid-5 |  |  |  |  | * |  |  |  |  |  |  |  |
| all | GMOY010152 | NTP |  |  |  | * |  |  |  |  |  |  |  |  |
| all | GMOY010173^§^ | NTP |  |  | ** |  |  |  |  |  | ** |  |  |  |
| all | GMOY010213 | DNA topoisomerase 2 |  |  | * |  |  |  |  |  | *** |  |  |  |
| all | GMOY010214 | NTP |  |  |  |  |  |  | * |  |  |  |  |  |
| all | GMOY010234 | alsin homolog |  |  |  |  | * |  |  |  |  |  |  |  |
| all | GMOY010310 | NTP |  |  |  |  |  | ** |  |  |  |  |  |  |
| all | GMOY010311 | NTP |  | * |  |  |  |  |  |  |  |  |  |  |
| all | GMOY010344 | NTP |  | * |  |  |  |  |  |  |  |  |  |  |
| all | GMOY010384 | NTP |  |  |  |  |  |  |  | *** |  |  |  |  |
| all | GMOY010447 | blue cheese |  |  |  |  | * |  |  |  |  |  |  |  |
| all | GMOY010651 | peanut |  |  |  |  |  |  |  |  |  |  | *** |  |
| all | GMOY010684 | scalloped | *** | *** |  |  |  |  |  |  |  |  |  |  |
| all | GMOY010944 | helicase domino | ** |  |  |  | *** |  |  |  | ** |  | ** |  |
| all | GMOY011064 | NTP |  |  | * |  | *** |  |  |  | * |  |  |  |
| all | GMOY011075 | NTP |  |  |  |  |  |  |  |  |  | * |  |  |
| all | GMOY011328 | NTP | *** | *** |  |  |  |  |  | * |  |  |  |  |
| all | GMOY011499 | NTP |  |  |  |  |  |  |  | ** |  |  |  |  |
| all | GMOY011575 | empty spiracles |  |  |  |  |  | * |  |  |  |  |  |  |
| all | GMOY011709 | NTP |  | ** |  |  |  |  |  |  |  |  |  |  |
| all | GMOY011918 | NTP |  |  |  |  | ** |  |  |  |  |  |  |  |
| all | GMOY011994 | discs overgrown |  |  |  |  | * |  |  |  |  |  |  |  |
| all | GMOY012004 | organic cation transporter |  |  | *** | *** | *** |  |  | *** |  |  |  | *** |
| Noaus | GMOY009305 | NTP |  |  |  |  |  |  |  |  |  |  |  | * |
| NOb | GMOY000017 | NTP |  |  |  |  |  |  |  |  | ** |  |  |  |
| NOb | GMOY000301 | structural maintenance of chromosomes |  |  |  | * |  |  |  |  |  |  |  |  |
| NOb | GMOY000392 | lodestar |  |  |  |  |  |  |  |  | *** |  |  |  |
| NOb | GMOY001122 | NTP |  |  | * |  | * |  |  |  | *** |  |  |  |
| NOb | GMOY002664 | NTP | *** | *** |  |  |  |  |  | ** |  |  |  |  |
| NOb | GMOY002933 | NTP |  | * |  |  |  |  |  |  |  |  |  |  |
| NOb | GMOY003749 | NTP | *** | *** |  |  |  |  |  |  |  |  |  |  |
| NOb | GMOY003818 | NTP |  |  |  | * |  |  |  |  |  |  |  |  |
| NOb | GMOY005118 | Major Facilitator Superfamily Transporter |  |  |  |  | * |  |  |  |  |  |  |  |
| NOb | GMOY005278 | NTP |  |  |  |  |  |  |  |  |  | * |  |  |
| NOb | GMOY005825 | NTP |  |  |  |  | * |  |  |  |  |  |  |  |
| NOb | GMOY006700 | lamin | * | * |  |  |  |  |  |  |  |  |  |  |
| NOb | GMOY007135 | pellino | *** | *** | *** |  | *** |  | *** |  | *** |  | *** |  |
| NOb | GMOY007250 | NTP |  |  |  |  | * |  |  |  |  |  |  |  |
| NOb | GMOY007798 | E3 ubiquitin-protein ligase | ** | *** |  |  |  |  |  |  |  |  |  |  |
| NOb | GMOY007904 | sevenless |  |  |  |  |  |  |  | ** |  |  |  |  |
| NOb | GMOY007927 | Beta-hexosaminidase |  | * |  |  |  |  |  |  |  |  |  |  |
| NOb | GMOY009121 | ecdysone receptor |  |  | * |  |  |  |  |  |  |  |  |  |
| NOb | GMOY010705 | NTP | *** | *** |  |  |  |  |  |  |  |  |  |  |
| NOb | GMOY010738 | NTP |  |  |  |  |  | ** |  |  |  |  |  |  |
| NOb | GMOY010740 | Deubiquitinating apoptotic inhibitor |  |  | * |  |  |  |  |  |  |  |  |  |
| NOb | GMOY010796 | NTP |  | * |  |  |  |  |  |  |  |  |  |  |
| NOb | GMOY011696 | NTP |  |  |  |  |  |  |  |  |  | * |  |  |
| NOb | GMOY011858 | NTP |  |  |  |  |  |  |  | * |  |  |  |  |
| NOf | GMOY000269 | sugar transporter | *** | *** |  |  | ** |  |  |  |  | * |  |  |
| NOf | GMOY010920 | NTP |  |  | *** |  |  |  |  |  |  |  |  |  |
| NOf | GMOY011611 | NTP |  |  |  |  |  |  |  |  |  |  | * |  |
| NOpai | GMOY002453 | NTP |  |  |  |  | * |  |  |  |  |  |  |  |
| NOpai | GMOY003306 | protein TsetseEP |  |  |  |  |  |  |  |  |  |  | ** |  |
| NOpai | GMOY004000 | NTP |  |  |  |  |  |  | *** |  |  |  | ** | * |
| NOpai | GMOY007507 | Elongin B |  |  |  | * |  |  |  |  |  |  |  |  |
| NOpai | GMOY008607 | NTP | *** | *** |  |  |  |  |  |  |  |  |  |  |
| NOppi | GMOY002073 | NTP | *** | *** |  |  |  |  |  |  |  | * |  |  |
| NOppi | GMOY003159 | NTP |  |  | * |  |  |  |  |  |  |  |  |  |
| NOppi | GMOY004902 | HIB CoA deacylase |  |  |  |  |  |  |  |  | *** |  |  |  |
| NOppi | GMOY006151 | NTP | *** | *** |  |  |  |  |  |  |  |  |  |  |
| NOppi | GMOY008445 | 1-phosphatidylinositol-3-phosphate 5-kinase |  |  |  |  | * |  |  |  |  |  |  |  |
