# Supplementary material for: Viviparity and habitat restrictions may influence the evolution of male reproductive genes in tsetse fly (Glossina) species
Source: BMC Biol. 2021 Sep 23;19:211. doi: 10.1186/s12915-021-01148-4 (PMC8461966; doi:10.1186/s12915-021-01148-4)
Supplement: Supplementary file 5 — Additional file 5: Table S5. Number of MAG genes (n = 10) in G. m. morsitans, G. austeni, G. fuscipes, G. pallidipes and G. palpalis tested for positive selection after site (A and B), branch (Br) and branch-site (BrS) models. Dataset column indicates the number of species in which ortholog sequences of each G. m. morsitans gene were identified (b = G. brevipalpis; ppi = G. palpalis). Gene ID column reports G. m. morsitans orthologs. NTP indicate genes encoding Novel Tsetse Proteins. * FDR < 0.20; ** FDR < 0.05; *** FDR < 0.005. [file 12915_2021_1148_MOESM5_ESM.docx]

**Supplementary Table 5. Number of MAG genes (*n* = 10) in *G. m. morsitans, G. austeni*, *G*. *fuscipes*, *G. pallidipes* and *G. palpalis* tested for positive selection after site (A and B), branch (Br) and branch-site (BrS) models.** Dataset column indicates the number of species in which ortholog sequences of each *G. m. morsitans* gene were identified (b=*G. brevipalpis*; ppi=*G. palpalis*). Gene ID column reports *G. m. morsitans* orthologs. NTP indicate genes encoding Novel Tsetse Proteins. * FDR < 0.20; ** FDR < 0.05; *** FDR < 0.005. ^§^Genes encoding spermatophore proteins in *G. m. morsitans* (Scolari et al., 2016).

|  |  |  | **Site test** | | ***G.morsitans*** | | ***G.austeni*** | | ***G.fuscipes*** | | ***G.pallidipes*** | | ***G.palpalis*** | |
| --- | --- | --- | --- | --- | --- | --- | --- | --- | --- | --- | --- | --- | --- | --- |
| **Dataset** | **Gene ID** | **Putative Function** | **A** | **B** | **Br** | **BrS** | **Br** | **BrS** | **Br** | **BrS** | **Br** | **BrS** | **Br** | **BrS** |
| all | GMOY002550^§^ | deoxyribonuclease II |  |  | ** |  |  |  |  |  |  |  |  |  |
| all | GMOY002583 | NTP |  |  |  |  |  |  |  |  |  | * |  |  |
| all | GMOY005874^§^ | OBP |  |  |  | * |  |  |  |  |  |  |  |  |
| all | GMOY005914 | transmembrane channel 1 |  |  |  |  |  |  |  | * |  |  |  |  |
| all | GMOY007314^§^ | odorant binding protein 17 | ** | ** |  |  |  |  |  |  |  |  |  |  |
| NOb | GMOY002399^§^ | NTP | *** | *** |  |  | * | * |  | ** |  |  |  |  |
| NOb | GMOY004505^§^ | NTP |  |  |  | * |  |  |  |  |  |  |  |  |
| NOb | GMOY007759 | NTP | *** | *** |  |  |  |  |  |  |  |  |  |  |
| NOb | GMOY010622 | synaptic vesicular amine transporter isoform X1 |  |  |  |  |  | * |  |  |  |  | * |  |
| NOppi | GMOY009723^§^ | angiotensin-converting enzyme-related |  |  |  |  | * |  |  |  |  |  |  |  |
