# Supplementary material for: Viviparity and habitat restrictions may influence the evolution of male reproductive genes in tsetse fly (Glossina) species
Source: BMC Biol. 2021 Sep 23;19:211. doi: 10.1186/s12915-021-01148-4 (PMC8461966; doi:10.1186/s12915-021-01148-4)
Supplement: Supplementary file 6 — Additional file 6: Table S6. List of species, strains and genome resources used in this study. [file 12915_2021_1148_MOESM6_ESM.docx]

**Supplementary Table 6. List of species, strains and genome resources used in this study.**

| Genus | Species | Strain | Genome assembly |  |
| --- | --- | --- | --- | --- |
| *Glossina* |  |  |  |  |
|  | *G. m. morsitans* | Yale | GmorY1.5 |  |
|  | *G. pallidipes* | IAEA | GpaII1.3 |  |
|  | *G. austeni* | TTRI | GausT1.3 |  |
|  | *G. fuscipes* | IAEA | GfusI1.3 |  |
|  | *G. palpalis* | IAEA | GpapI1.1 |  |
|  | *G. brevipalpis* | IAEA | GbreI1.3 |  |
| *Drosophila* |  |  |  |  |
|  | *D. melanogaster* | iso-1 | Dmel_6.14 |  |
|  | *D. grimshawi* | Tucson 15287-2541.00 | dgri_caf1 |  |
|  | *D. ananassae* | Tucson 14024-0371.13 | dana_caf1 |  |
| *Musca* |  |  |  |  |
|  | *M. domestica* | Aabys | MdomA1 |  |
| *Lutzomyia* |  |  |  |  |
|  | *L.* *longipalpis* | Jacobina | LIonJ1.2 |  |
| *Anopheles* |  |  |  |  |
|  | *A. gambiae* | PEST | AgamP4.4 |  |
